# Urea level is an independent predictor of mortality in patients with severe aortic valve stenosis

**DOI:** 10.1371/journal.pone.0230002

**Published:** 2020-03-11

**Authors:** Dan Haberman, Gil Chernin, Valery Meledin, Meital Zikry, Mony Shuvy, Gera Gandelman, Sorel Goland, Jacob George, Sara Shimoni

**Affiliations:** 1 The Heart Institute, Kaplan Medical Center, Rehovot, Israel; 2 Hebrew University and Hadassah Medical School, Jerusalem, Israel; 3 Nephrology Department Kaplan Medical Center, Rehovot, Israel; 4 Heart Institute, Hadassah Medical Center, Jerusalem, Israel; Boston University, UNITED STATES

## Abstract

**Introduction:**

Severe aortic stenosis (AS) is the most common valvular heart disease in the western world. Various factors are related to severe AS prognosis, including chronic kidney disease. The aim of this study was to evaluate the prognostic value of urea level in patients with severe AS.

**Methods:**

We prospectively enrolled 142 patients (79.1±9.4 years, 88 women) with severe AS (mean valve area 0.67± 0.17 cm^2^). Clinical assessment, blood tests and echocardiography were performed at enrollment and follow up. The patient population was divided into low and high urea level groups, according to the median urea level at enrollment (72 patients, mean urea 35.5±6.2 mg/dL and 70 patients, mean urea 61.1±17.8 mg/dL, respectively). Hundred and twelve patients (79%) underwent aortic valve intervention. The primary endpoint was all-cause and cardiovascular mortality.

**Outcomes:**

During follow-up of 37±19.5 months, 56 (37.1%) patients died, 39 due to cardiovascular causes. In univariate analysis, age, urea level, creatinine, New York Heart Association (NYHA) class and aortic valve intervention were associated with all-cause mortality. However, in multivariate analysis only aortic valve intervention and blood urea were independent predictors of all-cause mortality (HR 0.494; 95% CI 0.226–0.918, P = 0.026 and HR 1.015; 95% CI 1.003–1.029, P = 0.046 respectively). Urea level, NYHA class and age were also significant predictors of cardiovascular mortality. Whereas, in multivariate analysis, only urea level predicted cardiovascular mortality in these patients (HR 1.017; CI 1.003–1.031 P = 0.019).

**Conclusions:**

Blood urea, a generally readily available and routinely determined marker of renal function, is an independent prognostic factor in patients with severe AS.

## Introduction

Aortic Stenosis (AS) is the most common valvular heart disease in the western world. [1] AS is characterized by progressive narrowing of the valve orifice due to an active inflammatory and potentially modifiable process, with similarities to atherosclerosis.[2, 3]

AS predominance increases with age and constitute a significant cause for morbidity and mortality in elderly patients. Aortic valve stenosis is the primary indication for valve replacement in western countries, and the number of interventions continues to increase as the population grows older.

Various risk factors are associated with the development and progression of aortic valve stenosis. These include hypertension, diabetes, hyperlipidemia and obesity.[4] Chronic kidney disease (CKD) is another risk factor for AS. Left-sided valve disease is highly prevalent and associated with higher mortality among patients CKD.[5]

The prognosis of AS mainly depends on the clinical course, as patients can remain asymptomatic for many years due to compensatory mechanisms of left ventricle hypertrophy which normalizes wall tension and maintains cardiac output.[6] However with time, this compensatory mechanism may fail and lead to irreversible myocardial injury and fibrosis.

The traditional patient assessment is focused on the severity of the aortic stenosis and patient symptoms, with limited ability to predict the time of symptom onset or the likelihood of clinical deterioration for a given patient.

Various biomarkers have been an area of ongoing interest in AS. B-type natriuretic peptide (BNP) was shown to proceed symptoms development in patients with AS and predict prognosis [7–9] and indeed, BNP levels are included in clinical guideline for AVR in asymptomatic AS patients and low surgical risk. [10] Measurement of biomarkers in patients with AS could potentially be useful to minimize morbidity and mortality before and after valve replacement and to optimize the time of valve replacement. Biomarkers can identify higher-risk subgroups that may need more careful follow-up before and after valve replacement to minimize heart failure symptoms and hospitalization.

In heart failure patients presenting with acute decompensated heart failure blood urea nitrogen (BUN), BNP and low diastolic blood pressure where shown to predict cardiovascular morbidity and mortality.[11] We aimed to study the predictive value of urea level on the prognosis of patients with severe AS.

## Methods

The study prospectively included 152 patients with severe AS diagnosed by echocardiography who were followed in the valvular disease clinic in Kaplan Medical Center (Rehovot, Israel) between November 2010 and July 2013. Ten patients were excluded due to incomplete clinical data and follow up. This study was approved by the Kaplan Medical Center institutional ethics committee and all patients provided written informed consent.

Patient population was divided into two groups based on the median urea value, 43 mg/dL. The low urea level group included 72 patients with mean urea level of 35.5±6.2 mg/dL and high urea level group with 70 patients with mean urea level of 61.1±17.8 mg/dL.

We collected the following information: patient demographic data, medical history, current medication, clinical and echocardiographic findings and clinical outcomes. Coronary artery disease (CAD) was defined by one of the following: 1. Coronary stenosis of more than 70% on coronary angiogram or Computer Tomography, 2. History of myocardial infarction or previous revascularization.

We obtained fasting blood sample for measurement of glucose, urea, creatinine, aspartate aminotransferase, alanine aminotransferase, uric acid, total bilirubin, sodium and potassium.

Glomerular filtration rate (GRF) was calculated according to CKD-EPI formula.

### Follow up

Patients were followed in valvular heart disease clinic in Kaplan Medical Center on 6-months clinic visit basis. The decision to preform aortic valve replacement (AVR) was done by the cardiologist based on patients' symptoms, echocardiographic data and patients’ risk, according to clinical guidelines.

The primary endpoint was all-cause and cardiac mortality. Causes of death were determined by examination of hospital records and medical files of patients' general practitioners. Deaths due to cardiovascular causes included sudden deaths and deaths from acute myocardial infarction (MI), cerebrovascular accident (CVA) or congestive heart failure (CHF).

### Echocardiography studies

Transthoracic echocardiography including assessment of the aortic valve was performed according to established guidelines [12, 13]. Left ventricular dimensions were assessed in 2D images and left ventricular ejection fraction (LVEF) was measured using modified Simpson's method. Mean and peak aortic valves gradients were measured and aortic valve area was calculated using the continuity equation. The severity of AS was defined based on various parameters as indicated in the guidelines [12, 13]. Mild, moderate and severe AS was defined as valve area of 1.5–2.0 cm2, 1.0–1.5 cm2 and less than 1.0 cm2, respectively. Diastolic dysfunction was evaluated according to established guidelines.[14]

### Statistical analysis

Results are expressed as the mean ±SD or as percentages. Student’s t-test was used to compare differences between groups for continuous variables and the chi-square test was used for categorical data. The association between clinically relevant variables and event free survival (cardiovascular mortality and all-cause mortality) was assessed using univariate analysis. For categorical variables, a Kaplan-Meier survival analysis was used to plot event free survival with the log-rank test to compare the survival plots. To test the association between continuous variables and event free survival, Cox regression for survival analysis was utilized. The variables that were found to be significant in univariate analysis were introduced into a multivariate Cox proportional hazards regression model. Since the event rate of cardiac mortality was lower compared to all-cause mortality, we used Cox proportional hazards regression model using the stepwise, forward, likelihood ratio method for variable selection out of the potential variables. The hazard ratio (HR) and 95% confidential interval (CI) were calculated. The impact of AVR during follow-up was tested with AVR as a time-dependent covariate in the stratified Cox proportional hazards model for overall and cardiovascular survival. All statistical analyses were performed using commercially available software (SPSS v22). All tests were bilateral and p value <0.05 was considered significant.

## Results

### Patient population

The final study population included 142 patients with severe aortic stenosis. Patients were divided into two groups based on the median urea value. Low urea levels group with urea ≤43 mg/dL and high urea levels group with urea >43 mg/dL. Baseline characteristics are listed in Table 1. Patients in high urea levels group were older, where of lower NYHA class, higher creatinine levels, lower GFR and higher uric acid levels. Patients in high urea level group had more frequent history of coronary artery disease and higher left ventricle septal and posterior wall thickness. However, there were no differences in left ventricular mass, and no differences in other echocardiographic parameters including left ventricular function, diastolic dysfunction grade, mitral regurgitation severity and aortic stenosis severity. The two groups were similar with regards to gender, risk factors, medical therapy and procedural therapy (Transcutaneous aortic valve implantation- TAVI or surgical aortic valve replacement-SAVR). Hemodynamic parameters including heart rate and systolic and diastolic blood pressure did not differ significantly between the groups.

**Table 1:** Severe aortic stenosis patient baseline characteristics.

	Parameter	All patients	Urea ≤ 43	Urea < 43	
			1	2	P value
	N	142	72	70	
**Demographics and physical**					
	Age (years)	**79.1** ± 9.4	**76.7** ± 10.6	**81.7** ± 7.3	**0.002**
	Male gender	**54** (38%)	**26** (36%)	**28** (40%)	0.73
	BMI	**27.8** ± 4.9	**27.7** ± 4.5	**28.0** ± 5.4	0.77
**Past medical history**					
	HTN	**115** (81%)	**55** (76%)	**60** (86%)	0.2
	DM	**51** (36%)	**22** (31%)	**29** (41%)	0.22
	Dyslipidemia	**100** (70%)	**49** (68%)	**51** (73%)	0.58
	Coronary disease	**70** (49%)	**28 (39%)**	**42 (60%)**	**0.012**
**Laboratory values**					
	Urea (mg/dL)	**48.1** ± 18.4	**35.5** ± 6.2	**61.1** ± 17.8	**0.0001**
	Urea / Cr	**48.3** ± 15.5	**44.5** ± 14.6	**52.1** ± 15.8	**0.003**
	Serum Cr (mg/dL)	**1.04** ± 0.36	**0.86** ± 0.24	**1.22** ± 0.36	**0.0001**
	GFR (ml/min)	**63.5** ± 21.4	**75.4** ± 18.3	**51.2** ± 17.0	**0.0001**
	Uric Acid (mg/dL)	**6.2** ± 1.8	**5.35** ± 1.32	**7.03** ± 1.83	**0.0001**
	Hg (g/L)	**12** ± 1.7	**12** ± 1.6	**12.1** ± 1.7	0.65
	Total Protein (g/L)	**6.8** ± 0.7	**6.8** ± 0.6	**6.9** ± 0.7	0.63
	Albumin (g/L)	**3.9** ± 0.4	**3.9** ± 0.4	**3.8** ± 0.4	0.79
	GOT (U/L)	**24.4** ± 10.3	**23.3** ± 8.6	**25.6** ± 11.8	0.19
	GPT (U/L)	**19.6** ± 10.3	**19.8** ± 10.8	**19.5** ± 9.9	0.88
	LDH (mmol/L)	**440** ± 128	**438** ± 106	**442** ± 146	0.88
**Clinical parameters**					
	NYHA				
	1 or 2	**60** (42%)	**38 (**53%)	**22** (31%)	0.011
	3 or 4	**82** (58%)	**34** (47%)	**48** (69%)	0.011
**Echo parameters**					
	LVDs (mm)	**27.1** ± 5.3	**26.7** ± 4.6	**27.6** ± 6.1	0.5
	LVDd (mm)	**45.9** ± 5.8	**46.8** ± 5.4	**45** ± 6	0.07
	LVEF (%)	**54.8** ± 7.2	**55.1** ±7.2	**54.4** ± 7.2	0.53
	AVA (cm^2^)	**0.68** ± 0.17	**0.69** ±0.16	**0.66** ± 0.18	0.33
	AS Grad, Peak (mmHg)	**0.68** ± 0.17	**76.8** ± 27.3	**77.2** ± 21.4	0.93
	AS Grad, Mean (mmHg)	**48.6** ± 16.2	**49.1** ± 17.2	**48.1** ± 15.2	0.71
	IVS thickness (mm)	**13.8** ± 2.0	**13.4** ± 1.8	**14.2** ± 2.1	**0.011**
	PS thickness (mm)	**12.3**± 1.5	**12** ± 1.4	**12.6** ± 1.6	**0.029**
	LV mass (g)	**236** ± 62.1	**234** ± 58.4	**239** ± 66.0	0.63
	PA pressure (mmHg)	**41.1** ± 12.7	**38.9** ± 12.0	**43.2** ± 13.2	0.08
	MR grade				0.76
	• Mild	**96** (68%)	**49** (68%)	**47** (67%)	
	• Moderate	**45 (**32%)	**22** (31%)	**23** (33%)	
	• Moderate to severe	**1** (<1%)	**1** (1%)	**0** (0%)	
	• Severe	**0**	**0**	**0**	
	DD grade				0.19
	• Grade I	**104** (73%)	**56** (78%)	**48** (69%)	
	• Grade II	**29** (21%)	**12** (17%)	**17** (24%)	
	• Grade III	**9** (6%)	**4** (5%)	**5** (7%)	
**Hemodynamic parameters**					
	Heart rate (bpm)	**69** ± 11	**68** ± 10	**71** ± 12	0.13
	SBP (mmHg)	**126** ± 15	**124** ± 12	**128** ± 17	0.11
	DBP (mmHg)	**66** ± 11	**65** ± 11	**67** ± 12	0.18
**Medical treatment**					
	ACE-I	**75** (53%)	**36** (50%)	**39** (56%)	0.507
	ARB	**26** (18%)	**15** (21%)	**11** (16%)	0.517
	B-Blockers	**94** (66%)	**44** (61%)	**50** (71%)	0.217
	Furosemide	**41** (29%)	**16** (14%)	**25** (36%)	0.096
	Spironolactone	**10** (7%)	**3** (4%)	**7** (10%)	0.205
	Digoxin	**15** (10%)	**8** (11%)	**6** (9%)	0.78
	Nitrates	**15** (11%)	**7** (10%)	**8** (11%)	0.79
**Intervention**					
	AVR	**45** (32%)	**28** (39%)	**17** (24%)	0.07
	TAVI	**67** (47%)	**32** (44%)	**35** (50%)	0.61

Abbreviations

HTN, hypertension; DM, diabetes; Cr, creatinine; GFR, glomerular filtration rate; Hg, hemoglobin; GOT, glutamic oxaloacetic transaminase; GPT, glutamic pyruvic transaminase; NYHA, New York heart association classification; LVDs/d, left ventricle diameter systole/diastole; LVEF, left ventricle ejection fraction; AS Grad, aortic stenosis gradient; IVS, inter ventricular septum; PS, posterior wall; PA, pulmonary artery; MR, mitral regurgitation; DD, diastolic dysfunction; ACE-I, Angiotensin converting enzyme inhibitor; ARB, Angiotensin II receptor blocker; AVR, aortic valve replacement; TAVI, transcatheter aortic valve implantation.

Liver enzyme along with serum albumin and total protein were within normal range in our cohort.

Catabolic state can cause high urea levels as well. BMI, GOT, total protein and albumin were within normal range in both groups and did not differ between the two groups, suggesting that no significant long term catabolism was present in our patient population.

### Outcomes/follow up

During a mean follow up of 37±19.5 months, 56 (39.4%) patients died, out of which 37 (26.1%) were cardiovascular-related deaths. Cardiovascular mortality was due to heart failure in 17 (46%) patients, acute MI in 2 (5.4%), CVA in 6 (16.2%) and sudden death in 12 (32.4%). The causes of non-cardiovascular mortality were mainly due to infections disease.

Table 2 shows the various parameters related to all-cause mortality.

**Table 2:** Predictors of all-cause mortality in patients with severe aortic stenosis.

		Univariate			Multivariate	
Parameter	Hazard Ratio	Confidence Interval	P Value	Hazard Ratio	Confidence Interval	P Value
Age (years)	1.057	1.021–1.095	**0.002**	1.034	0.991–1.079	0.119
Gender (Male)	1.154	0.511–1.472	0.597			
Urea (mg/dL)	1.023	1.012–1.034	**0.0001**	1.015	1.003–1.029	**0.046**
Urea / Cr	1.011	0.996–1.026	0.158			
Serum Cr	2.36	1.261–4.418	**0.007**			
Uric Acid (mg/dL)	1.101	0.946–1.281	0.215			
GFR	0.980	0.968–0.992	**0.001**	0.998	0.980–1.017	0.858
Total Protein	0.829	0.559–1.230	0.352			
HTN	0.979	0.529–1.937	0.949			
DM	1.146	0.506–1.504	0.623			
Coronary disease	1.660	0.975–2.829	0.065			
NYHA	1.582	1.096–2.284	**0.014**	1.410	0.949–2.094	0.089
NYHA 3–4 vs 1–2	1.976	1.117–3.498	**0.019**			
LVDd (mm)	0.98	0.933–1.029	0.416			
LVEF (est. %)	0.976	0.942–1.011	0.17			
AVA	0.321	0.063–1.645	0.173			
IVS (mm)	1.135	0.960–1.342	0.139			
PW (mm)	1.073	0.948–1.121	0.265			
ACE-I	0.706	0.834–2.407	0.198			
ARB	1.733	0.262–1.273	0.173			
B-Blockers	1.114	0.642–1.932	0.701			
Furosemide	0.917	0.618–1.924	0.766			
Intervention (TAVI or AVR), time dependent	0.485	0.269–0.873	**0.016**	0.494	0.226–0.918	**0.026**

Abbreviations

HTN, hypertension; DM, diabetes; Cr, creatinine; GFR, glomerular filtration rate; NYHA, New York heart association classification; LVDd, left ventricle diameter diastole; LVEF, left ventricle ejection fraction; AVA, aortic valve area; IVS, inter ventricular septum; PS, posterior wall; ACE-I, Angiotensin converting enzyme inhibitor; ARB, Angiotensin II receptor blocker

AVR, aortic valve replacement; TAVI, transcatheter aortic valve implantation.

Age, HYNA class, valve intervention, urea level, creatinine and GFR were significantly associated with increased mortality in univariate model. When the significant factors were tested in Cox Proportional Hazards Regression hazard model only urea level and intervention as a time dependent variable were significant predictor of mortality respectively (HR 1.015; CI 1.003–1.029, P = 0.046, HR 0.494; CI 0.226–0.918; P = 0.026). Since GRF and creatinine are strongly associated, only GFR was included in the model. When solely urea level, as a renal function parameter was entered into the model that also included age, NYHA class and intervention, urea level and intervention were still the only predictors of mortality, *w*ith a significantly lower p value for urea (HR 1.016; CI 1.004–1.027 P = 0.008, HR 0.496; CI 0.268–0.920; P = 0.026).

Urea level, NYHA class and age were also significant predictors of cardiovascular mortality, as shown in Table 3. In stepwise, forward, likelihood ratio Cox Proportional Hazards method for variable selection, only urea level predicted cardiac mortality in these patients (HR 1.017; CI 1.003–1.031 P = 0.019. NYHA class was not a significant predictor of cardiovascular mortality in multivariate model after correcting for other factors.

**Table 3:** Severe aortic stenosis cox proportional hazard (cardiac mortality).

		Univariate			Multivariate	
Parameter	Hazard Ratio	Confidence Interval	P Value	Hazard Ratio	Confidence Interval	P Value
Age (years)1.0471.004–1.092**0.033**Gender (Male)	1.085	0.552–2.131	0.813			
Urea (mg/dL)	1.017	1.003–1.031	**0.019**	**1.017**	**1.003–1.031**	**0.019**
Urea / Cr	1.009	0.990–1.029	0.351			
Serum Cr	1.751	0.778–3.943	0.176			
Uric Acid (mg/dL)	1.008	0.831–1.224	0.932			
GFR	0.986	0.972–1.001	**0.072**			
Total Protein	0.701	0.431–1.140	0.152			
HTN	0.905	0.413–1.980	0.802			
DM	0.681	0.336–1.379	0.286			
Coronary disease	1.874	0.961–3.656	0.065			
NYHA	1.642	1.041–2.589	**0.033**			
NYHA 3–4 vs 1–2	2.299	1.109–4.766	**0.025**			
LVDd (mm)	0.986	0.929–1.047	0.650			
LVEF (est. %)	0.969	0.929–1.011	0.149			
AVA	0.389	0.051–2.950	0.361			
ACE-I	1.215	0.633–2.331	0.559			
ARB	0.367	0.113–1.196	0.096			
B-Blockers	1.714	0.809–3.634	0.142			
Furosemide	1.217	0.611–2.424	0.576			
Intervention (TAVI or AVR), time dependent	0.720	0.354–1.464	0.364			

Abbreviations

HTN, hypertension; DM, diabetes; Cr, creatinine; GFR, glomerular filtration rate; NYHA, New York heart association classification; LVDd, left ventricle diameter diastole; LVEF, left ventricle ejection fraction; AVA, aortic valve area; ACE-I, Angiotensin converting enzyme inhibitor; ARB, Angiotensin II receptor blocker

AVR, aortic valve replacement; TAVI, transcatheter aortic valve implantation.

**Fig 1:**
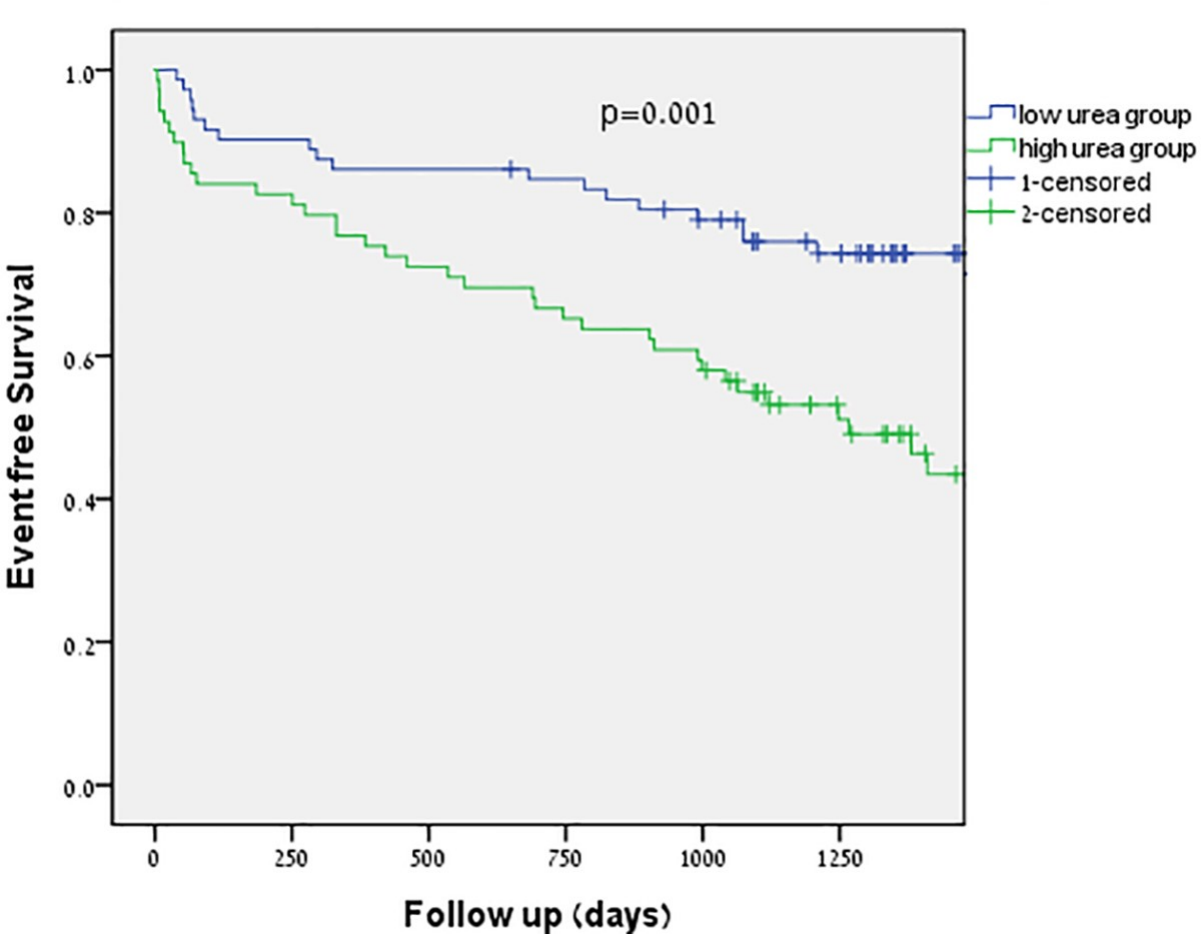
Survival curves in patients with severe aortic stenosis and low and high urea level.

**Fig 2:**
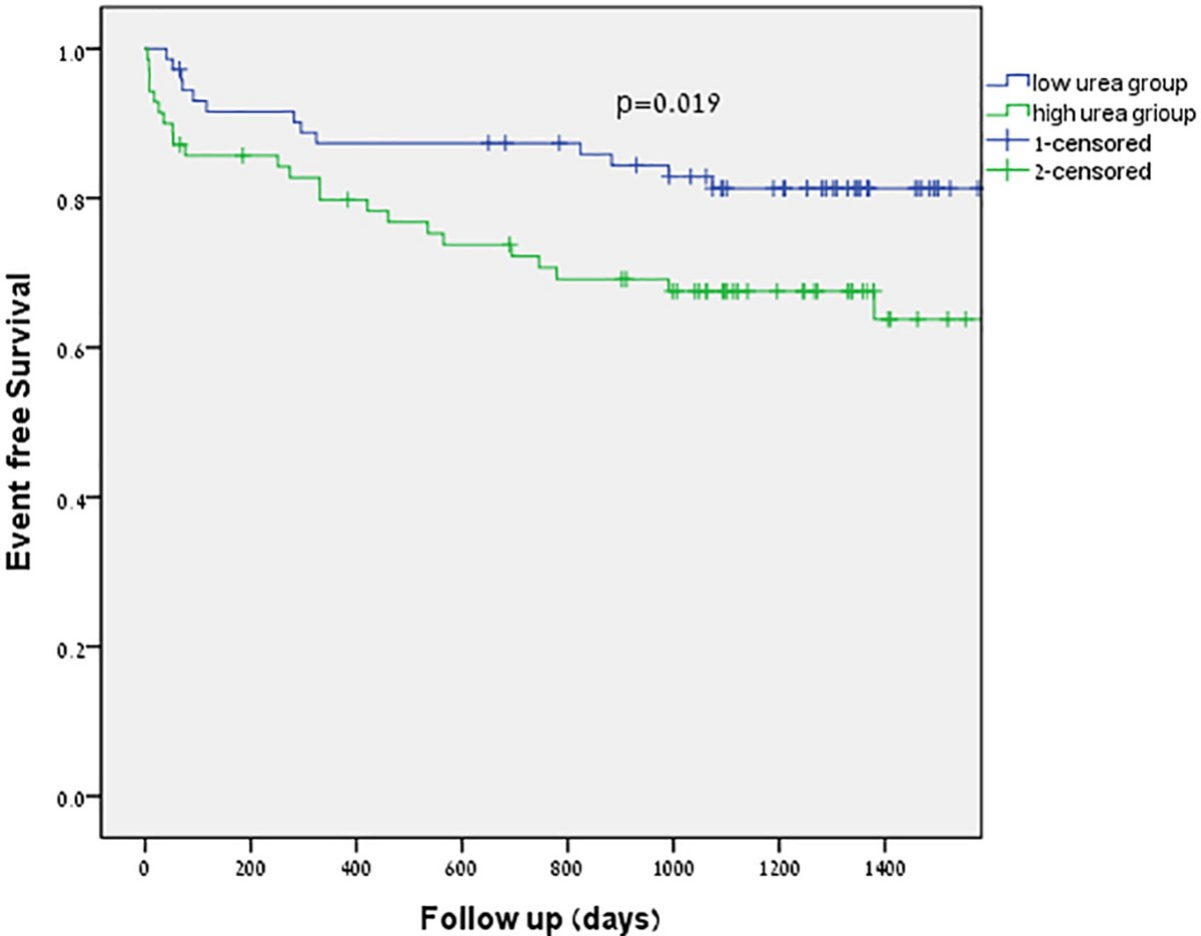
Cardiac event free survival in patients with severe aortic stenosis and low and high urea level.

Figs 1 and 2 show the Kaplan-Meier survival curves for all cause survival and cardiovascular event free survival in patients with severe AS. Patients in the low urea level group had significantly better survival rates compared to those in the high urea level group. This was the case for both all-cause mortality and cardiovascular mortality (p = 0.001 and p = 0.019 respectively).

## Discussion

In this study we demonstrated that in patients with severe aortic valve stenosis, urea level is a predictor of cardiovascular and all-cause mortality. The other known predictor of all-cause mortality was aortic valve replacement, whereas NYHA function class showed borderline significance in multivariate analysis. Urea level was more significant than GFR or creatinine measurements. To our knowledge this is the first study to show this correlation.

Renal dysfunction is linked to AS.[15] Renal impairment is associated with disease progression and prognosis in aortic stenosis. Chronic kidney disease accelerates the calcification of aortic valve in multiple pathways including altered mineral metabolism, inflammation, oxidative stress and hemodynamic overload.[16] Patients with early stage renal impairment have increased prevalence of AS in populations at high risk for coronary atherosclerosis. [17] Masuda et al showed that the prevalence of AS is higher in patients with CKD, and suggested that even small changes in GFR during initial stages of CKD can enhance the progression of aortic valve calcification in patients with high risk for coronary atherosclerosis. Recently, Vavilis et al showed that AS development is related to renal disease[18]. Interestingly, this association was less significant in patients with risk factors, because of common risk factors for both conditions such as diabetes mellitus and hypertension. Inflammation is an important process in aortic stenosis development and various studies showed correlation between CRP levels and other inflammatory markers and aortic stenosis in patients with CKD[15]. In addition, uncommon causes of AS such as systemic lupus erythematosus and Fabry's may also cause CKD.

In patients with end-stage renal disease and in patients on dialysis the association with calcific cardiovascular disease is even stronger.

CKD is also related to prognosis after cardiovascular and aortic valve procedures. [19] This is more significant in advanced stages of renal disease, with renal function being a significant determinant of midterm survival in patients undergoing either SAVR or TAVI. [20] CKD stages 3b to 5 have increased mortality after either TAVI or SAVR compared with patients with CKD stages 1 to 3a. However, there was no association between CKD and mortality in low- to intermediate-risk patients.

In our study population, the majority of the patients had normal or mild renal function impairment and moreover, the urea level was a better predictor of all-cause and cardiovascular mortality as compared to GFR and creatinine. This may suggest that the prognostic role of urea level in these patients is beyond renal function.

A high level of serum urea, along with BNP, is a well-known factor associated with increased mortality and hospitalizations rate in patients with heart failure. [21] The level of urea in the plasma represents the balance between production, excretion and reabsorption. The level of urea increases in heart failure via several mechanisms. There is reduced urea secretion and increased urea reabsorption. [22, 23] Low cardiac output leads to renin-angiotensin-aldosterone (RAAS) system and sympathetic nervous system (SNS) activation that causes reduction in GFR and lower urea secretion. RAAS activation increases urea absorption in the proximal tubule and the SNS increases urea absorption in the distal tubule. Vascular low hydrostatic pressure causes secretion of arginine vasopressin which increases urea reabsorption in the collecting duct. In addition, there is an increased hormonal catabolic stimulus that causes muscle protein breakdown, muscle wasting, increased amino acids release and consequent high level of urea in plasma. [24] All the above mechanisms lead to changes in urea levels. This demonstrates that urea does not only signifies low GFR, but also serves as complex metabolic, hormonal and hemodynamic biomarker. [25] In patients with heart failure, urea levels predicted prognosis mainly in patients with acute or decompensated heart failure. However, the levels of BUN predicted post discharge prognosis as well. [26] BUN levels were also shown to predict prognosis in patients with unstable and stable coronary disease, irrespective of renal function.[27, 28] In patients with AS, the disease of the valve leads to the disease of the myocardium. [29] The development of left ventricular hypertrophy (LVH) and fibrosis results in poor outcome in patients with AS, and the persistence of hypertrophy and diastolic dysfunction after surgical valve replacement increases mortality.[30, 31] The renin-angiotensin system is activated at an early stage in AS, promoting development of LVH, diastolic dysfunction, myocardial fibrosis and myocardial contractile failure. [32] In some observational studies, angiotensin-converting enzyme inhibitor and angiotensin-receptor blockers therapy have been shown to delay progression of AS and improve outcome in patients with AS. [33, 34]

Echocardiography is a gold standard for diagnosis and evaluation of AS. However, the indication for intervention is mainly clinical and this may be challenging in elderly patients with comorbidities. More sensitive biomarkers are warranted to evaluate ventricular decomposition, especially in asymptomatic patients. A potentially complementary approach to clinical and echocardiographic evaluation is the biomarkers. Biomarkers have been studied extensively in atherosclerosis. Recently, a large amount of data is also gathered on the role of biomarkers in aortic stenosis. [35–37] NT-proBNP levels correlate with AS severity and echocardiographic markers of higher risk for adverse outcomes in AS.[38] ST2 has shown to correlate with AS severity, symptoms and prognosis. [39–41] Other biomarkers, including hs-cTnT, Galectin-3, growth factors and models that include several biomarkers have shown to be associated with AS and correlate with prognosis.[39] Our results suggest that urea level, a simple and routinely used blood test, may serve as a useful biomarker in patients with AS.

### Limitations

The relatively small number of patients is one of the study limitations. However, the differences between the groups are significant. In addition, we did not assess BNP levels, therefore we could not assess the comparative prognostic significance of urea level compared to BNP. Another limitation is the fact that diuretic dose was not recorded. However, the use of diuretics did not differ significantly between the groups so this limitation does not appear to be significant.

## Conclusions

Blood urea, a generally readily available and routinely determined marker of renal function, is an independent prognostic factor in patients with severe AS. Urea level may serve as a complementary marker in asymptomatic patients before clinical symptoms arise. It may also shed light on the complex pathophysiological mechanism of hemodynamically significant aortic stenosis.

## Supporting information

S1 Data (XLSX)
